# Latent class growth modelling for the evaluation of intervention outcomes: example from a physical activity intervention

**DOI:** 10.1007/s10865-021-00216-y

**Published:** 2021-03-25

**Authors:** Anna-Maria Lampousi, Jette Möller, Yajun Liang, Daniel Berglind, Yvonne Forsell

**Affiliations:** 1 Department of Global Public Health, Karolinska Institutet, Stockholm, Sweden; 2 Institute of Environmental Medicine, Karolinska Institutet, Stockholm, Sweden

**Keywords:** Latent class growth analysis, LCGM, Trajectories, Intervention, Randomized trial, Physical activity

## Abstract

Intervention studies often assume that changes in an outcome are homogenous across the population, however this assumption might not always hold. This article describes how latent class growth modelling (LCGM) can be performed in intervention studies, using an empirical example, and discusses the challenges and potential implications of this method. The analysis included 110 young adults with mobility disability that had participated in a parallel randomized controlled trial and received either a mobile app program (n = 55) or a supervised health program (n = 55) for 12 weeks. The primary outcome was accelerometer measured moderate to vigorous physical activity (MVPA) levels in min/day assessed at baseline, 6 weeks, 12 weeks, and 1-year post intervention. The mean change of MVPA from baseline to 1-year was estimated using paired t-test. LCGM was performed to determine the trajectories of MVPA. Logistic regression models were used to identify potential predictors of trajectories. There was no significant difference between baseline and 1-year MVPA levels (4.8 min/day, 95% CI: −1.4, 10.9). Four MVPA trajectories, ‘Normal/Decrease’, ‘Normal/Increase’, ‘Normal/Rapid increase’, and ‘High/Increase’, were identified through LCGM. Individuals with younger age and higher baseline MVPA were more likely to have increasing trajectories of MVPA. LCGM uncovered hidden trajectories of physical activity that were not represented by the average pattern. This approach could provide significant insights when included in intervention studies. For higher accuracy it is recommended to include larger sample sizes.

**Electronic supplementary material:**

The online version of this article (10.1007/s10865-021-00216-y) contains supplementary material, which is available to authorized users.

## Introduction

The most common statistical approach for evaluating interventions is estimating the average change in pre-specified outcomes over time (Curran & Muthén, 1999). This approach is characterized as variable-centered and, although it can provide useful information about the effects of an intervention, it assumes that the change is homogenous within a study population (Jung & Wickrama, 2008; Muthén & Muthén, 2000). Such evaluations might be limited in capturing who benefited from the intervention and why, given that a population often consists of heterogenous groups regarding how they respond to a treatment or behavioral intervention (Falkenstein et al., 2019; Fitzpatrick et al., 2015). Taking a person-centered approach in the analysis, by identifying subgroups of individuals that follow the same pattern of change, could provide a more detailed insight on the effects of an intervention (Curran & Muthén, 1999; Muthen, 2002).

Latent class models are commonly used for identifying individuals with the same pattern of change regarding one or more outcomes, when repeated measurements are available (Andruff et al., 2009; van der Nest et al., 2020). Among these models is latent class growth modelling (LCGM), which in contrast to other methods does not require a priori knowledge about the number and shape of these trajectories (Andruff et al., 2009). Accordingly, different number and shape of trajectories can be tested and the model that fits the data better can be selected based on the combination of several parameters (Jung & Wickrama, 2008; Nagin, 1999). This approach can be used for exploratory purposes and uncover hidden trajectories within the population (van der Nest et al., 2020).

In the era of the obesity epidemic, LCGM could be useful for identifying individuals that do not respond to behaviour change interventions. A major risk factor for obesity is sedentary behavior, which refers to decreased energy expenditure (Matusitz & McCormick, 2012; Middelbeek & Breda, 2013). Individuals with mobility disability require special attention as they have lower physical activity levels compared to their able-bodied counterparts (Johnsen et al., 2017; Saebu & Sørensen, 2011). In addition, a bidirectional association has been observed between mobility disability and obesity (de Munter et al., 2016). High physical activity and cardiorespiratory fitness levels can support the attainment of a healthy body weight and prevent the negative consequences of sedentary lifestyle (McKinney et al., 2016). Previous studies report small to average effects of physical activity interventions on physical activity levels in people with mobility disability (Berglind et al., 2020; Ma & Martin Ginis, 2018). Identification of possible patterns of physical activity and their determinants might provide new evidence for the design of effective interventions for this population.

The use of latent class models for the evaluation of intervention outcomes has been proposed for almost 20 years (Curran & Muthén, 1999; Muthen, 2002). Although this method might provide important implications for future research, it is not commonly used in intervention studies. The objective of this article is to illustrate how LCGM can be performed in intervention studies and to discuss its potential challenges and implications. For this purpose, empirical data from a randomized controlled trial are analyzed, aiming to identify longitudinal patterns of moderate to vigorous physical activity (MVPA) and their determinants, among young adults with mobility disability.

## Methods

### Study design

The randomized controlled trial that was analyzed in this study has previously been described in detail (Berglind et al., 2018). In brief, it was a parallel trial including 110 individuals with mild mobility disability that were randomized to receive either a mobile app program (n = 55) or a supervised health program (n = 55). The duration of the intervention was 12 weeks and outcome measurements took place at baseline, 6 weeks, 12 weeks, and 1-year post intervention. In the current study, participants are observed through a longitudinal design and not analyzed as randomized.

### Study population

The eligibility criteria for participation in the trial was having self-reported mobility disability that was acquired over the past three years, age between 18 and 45 years, having access to a smartphone, and being able to speak and understand Swedish. Mobility disability was defined as having mobility limitations in daily activities, such as getting dressed or performing usual work tasks. Individuals requiring assistive devices or being unable to walk at a moderate to low intensity, were not included in the trial. Participants were recruited at private companies, rehabilitation and primary care centers in the Stockholm region, Sweden, during early 2018.

### Interventions

Detailed descriptions of the two interventions are presented in previous articles (Berglind et al., 2020; Lampousi et al., 2020). Both the mobile app program and the supervised health program involved the use of intrinsic motivation, self-monitoring, and goal setting techniques, which according to literature are effective in improving physical activity levels in adults (King et al., 2019). Participants in the mobile app program were encouraged to use the Acupedo walking app, a home-based training app that was developed by the Swedish Military (*Försvarsmaktens Träningsklubb*, n.d.), and the LogMyFood food photography app. The supervised group received personalized training at training facilities once per week for one hour and were also encouraged to exercise at home and walk daily. The training included both aerobic and strength exercises that were adapted to the baseline cardiorespiratory fitness levels of each individual. Participants in this group also received dietary advice and had access to the same food photography app as the mobile app group with some additional social network features.

### Data collection

#### Moderate to vigorous physical activity

The primary outcome of the randomized control trial was MVPA levels in minutes per day and was measured at all follow ups with the use of the Actigraph GT3X + accelerometers for seven consecutive days. Measurements were considered valid if participants had worn the device on their hip for at least 10 waking hours per day for a minimum of four days.

#### Cardiorespiratory fitness

Cardiorespiratory fitness was estimated with a submaximal cycle ergometer test (Ekblom-Bak test) (Björkman et al., 2016) at baseline and all follow up points as VO_2_max in ml/min/kg.

#### Demographic characteristics

Age and sex were self-reported at baseline through a web-based questionnaire.

#### Body mass index

Body mass index (BMI) was calculated as body weight in kilograms divided by the square of height in meters. Weight and height were measured at baseline by trained personnel with standardized equipment.

#### Physical functioning and bodily pain

Information on physical functioning and bodily pain was obtained at all examination points through the SF-36 questionnaire, which is a commonly used instrument for assessing health-related quality of life (Lins & Carvalho, 2016). The questionnaire consists of eight distinct domains, including physical functioning and bodily pain (Ware & Sherbourne, 1992). Each domain is scored from 0–100, with higher score indicating better functioning.

### Statistical analyses

After the trajectory analysis, logistic regression models were used in order to study the association between potential predictors of MVPA and trajectories. More specifically the odds ratios (OR) of having an improved pattern of MVPA were estimated in relation to age, sex, BMI, bodily pain, physical functioning, baseline MVPA and cardiorespiratory fitness levels, using the trajectory with the least improvement as reference group. All models that included MVPA and cardiorespiratory fitness were also adjusted for age.

All analyses were performed with Stata/IC 15. The multiple imputation approach using chained equations (MICE) was used for handling the missing data of MVPA at the three follow-up periods. The missing data were imputed using the predictive mean matching (PMM) method. According to this method, the imputed values were selected from the five closest to the predicted value observed values, based on a linear regression model. The selected number of imputations was 10. Possible predictors of MVPA at follow up and variables related to the missingness of MVPA (baseline MVPA, baseline VO_2_max, age, sex, physical functioning, bodily pain) were assessed through linear and logistic regression models respectively and were also included in the imputation model. Being assigned to the supervised group was related with lower odds of having missing MVPA observations at 1-year compared to the mobile app group (OR 0.28, 95% CI: 0.13, 0.61). Therefore, intervention group assignment was included in the multiple imputation model. Baseline characteristics were compared between those who attended the 1-year follow up and those who did not, using two-tailed t-tests for continuous variables and chi-squared tests for categorical variables (Electronic supplementary Table 1). Among the variables that were assessed as predictors of MVPA, baseline MVPA was associated with all follow up levels and was also included in the imputation model. The group level change in MVPA from baseline to 1-year in both observed and imputed values was estimated using two-tailed paired t-tests. A significance level of 0.05 was set for all statistical tests.

**Table 1:** Mean level of observed and imputed MVPA (min/day) at each time point by intervention group

MVPA (min/day)	Mean ± SD				Δ( 95% CI)
	Baseline	6 weeks	12 weeks	1 year	Baseline to 1 year^1^
*Observed*					
Total	44.3 ± 22.2	48.2 ± 16.5	44.3 ± 20.2	45.6 ± 23.7	4.8 (−1.4, 10.9)
Mobile app	48.4 ± 23.3	47.0 ± 14.4	43.6 ± 21.9	43.0 ± 26.8	−0.7 (−10.3, 8.9)
Supervised program	40.3 ± 20.6	49.3 ± 18.1	44.9 ± 19.0	47.0 ± 22.0	7.8 (−0.3, 15.8)
*Imputed*					
Total	44.4 ± 22.1	47.5 ± 15.7	43.9 ± 18.5	47.0 ± 19.1	2.6 (−1.5, 6.8)
Mobile app	48.4 ± 23.1	46.3 ± 13.6	43.0 ± 18.8	46.5 ± 19.2	−1.9 (−7.6, 3.8)
Supervised program	40.3 ± 20.6	48.6 ± 17.7	44.7 ± 18.4	47.6 ± 19.1	7.2 (1.2, 13.3)

^1^For the observed values n = 59 in the total sample, n = 21 in the mobile app program, n = 38 in the supervised health program; *MVPA* moderate to vigorous physical activity; *SD* standard deviation; *CI* confidence interval

LCGM was used for identifying the trajectories of MVPA from baseline, 6 weeks, 12 weeks to 1-year follow-up. Several models were fitted including different number of classes and polynomial forms (linear, quadratic, cubic) for each imputed dataset. All estimates and model parameters were pooled across the imputed datasets. The best model was selected based on the Bayesian information criteria (BIC), percentage of participants per class, and mean posterior probabilities of class membership. Better goodness of fit was indicated by the lowest BIC and higher posterior probabilities of class membership. The maximum number of classes that was included in the models was 5, since the goodness of fit started decreasing after considering more than four trajectories. In all models there were high posterior probabilities of group assignment (> 0.8) and more than 10% of participants in each class (Electronic supplementary Table 2). The model with the lowest BIC was the linear with three classes. However, the linear model with four classes had similar goodness of fit and was selected as the final model, due to the provision of distinct and more informative trajectories.

**Table 2:** Baseline characteristics of participants across the trajectories

Baseline characteristics	‘Normal/ Decrease’ (n = 31)	‘Normal/ Increase’ (n = 31)	‘Normal/Rapid Increase’ (n = 30)	‘High/Increase’ (n = 18)
Females, n (%)	27 (87)	24 (77)	24 (80)	15 (83)
Mobile app group, n (%)	19 (61)	12 (39)	14 (47)	10 (56)
Age (years), mean ± SD	38.8 ± 5.1	34.0 ± 5.9	34.9 ± 6.7	31.0 ± 5.5
BMI (kg/m^2^), mean ± SD	26.9 ± 5.5	26.4 ± 4.3	27.6 ± 6.4	25.6 ± 5.9
VO_2_ max (ml/kg/min), mean ± SD	32.6 ± 7.3	35.1 ± 7.1	36.4 ± 9.8	39.9 ± 7.5
MVPA (min/day), mean ± SD	26.8 ± 13.2	37.8 ± 10.6	50.9 ± 18.1	74.1 ± 21.3
Bodily pain (score), mean ± SD	55.5 ± 22.2	49.9 ± 21.7	47.4 ± 16.6	49.5 ± 15.3
Physical functioning (score), mean ± SD	76.0 ± 13.4	71.1 ± 15.8	70.7 ± 20.4	74.7 ± 15.7

*BMI* body mass index; *MVPA* moderate to vigorous physical activity; *SD* standard deviation

## Results

Table 1 shows the average MVPA at all follow ups for the observed and the imputed values. Those that attended all follow ups (n = 59) had an average MVPA of 40.8 min/day at baseline which increased to 45.6 min/day. However, this change was not statistically significant (4.8 min/day, 95% CI: −1.4, 10.9). Participants in the supervised program improved their MVPA levels (7.8 min/day, 95% CI: −0.3, 15.8), while the levels of those in the mobile app program remained stable (−0.7 min/day, 95% CI: −10.3, 15.8). In the imputed values, the difference of MVPA from baseline to 1-year in total participants was 2.6 min/day (95% CI: −1.5, 6.8) which was also not statistically significant.

**Fig. 1:**
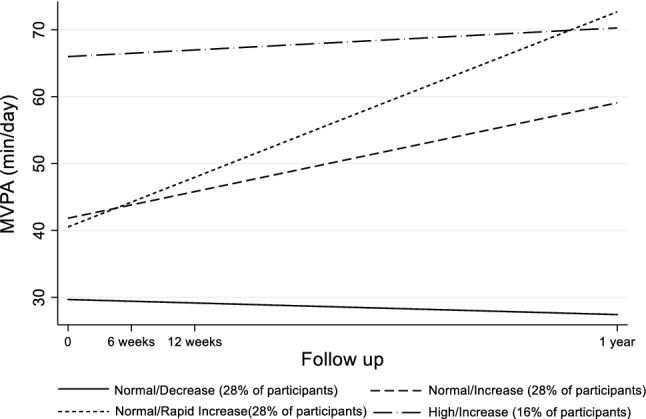
Trajectories of moderate to vigorous physical activity (min/day) at baseline, 6 weeks, 12 weeks and 1-year post intervention

Figure 1 illustrates the different trajectories that were identified. More specifically, the first group had normal baseline MVPA levels and followed a decreasing pattern ‘Normal/Decrease’, the second had normal baseline levels which increased over time ‘Normal/Increase’, the third had also normal baseline levels that increased more rapidly ‘Normal/Rapid increase’, and the fourth one had high baseline levels and followed an increasing pattern ‘High/Increase’. The ‘High/Increase’ trajectory included 16% of total participants, while each of the other three trajectories included 28%. The baseline characteristics of participants across the different classes are presented in Table 2. Most participants were females (90/110) and were represented in high proportions at all classes. Those in the ‘Normal/Decrease’ class had the highest mean age and lowest baseline MVPA, while those in the ‘High/Increase’ class had the lowest mean age and highest baseline MVPA. All classes were represented by moderate levels of bodily pain and good physical functioning at baseline.

### Predictors of trajectories

With increasing age, the odds of being in the ‘Normal/Increase’, ‘Normal/Rapid Increase’, and ‘High/Increase’ trajectories were lower compared to the ‘Normal/Decrease’ trajectory (see Table 3). Whereas, with the increase of baseline MVPA, the odds of being in any increasing trajectory were higher than being in the ‘Normal/Decrease’ trajectory. The highest odds in relation to MVPA were observed for the ‘High/Increase’ trajectory (OR 1.31, 95% CI: 1.04, 1.64). Baseline MVPA remained a predictor of class assignment in all models, after adjusting for age (results not shown). VO_2_max was associated with higher odds of being in the ‘High/Increase’ trajectory (OR 1.14, 95% CI: 1.04, 1.64). This association did not remain statistically significant after adjusting for age (OR 1.10, 95% CI: 0.99, 1.23).

**Table 3:** Crude odds ratios and 95% confidence intervals of the ‘Normal/Increase’, ‘Normal/Rapid Increase’, and ‘High/Increase’ trajectories of MVPA compared to the ‘Normal/Decrease’ trajectory, in relation to baseline characteristics. Results from logistic regression models

	‘Normal/Increase’	‘Normal/Rapid Increase’	‘High/Increase’
Baseline characteristics	OR (95% CI)^1^	OR (95% CI)^1^	OR (95% CI)^1^
Age (years)	0.85 (0.77, 0.95)	0.90 (0.82, 0.98)	0.78 (0.67, 0.89)
Sex (Male)	1.97 (0.51, 7.56)	1.69 (0.42, 6.70)	1.35 (0.27, 6.85)
BMI (kg/m^2^)	0.98 (0.88, 1.08)	1.01 (0.93, 1.11)	0.95 (0.85, 1.07)
Bodily pain (score)	0.99 (0.97, 1.01)	0.98 (0.95, 1.00)	0.98 (0.95, 1.06)
Physical functioning	0.98 (0.94, 1.01)	0.98 (0.95, 1.01)	0.99 (0.95, 1.04)
VO_2_ max (ml/kg/min)	1.05 (0.98, 1.13)	1.06 (0.99, 1.12)	1.14 (1.04, 1.26)
MVPA (min/day)	1.08 (1.03, 1.13)	1.10 (1.05, 1.15)	1.31 (1.04, 1.64)

^1^Reference group: ‘Normal/Decrease’ trajectory; *BMI* body mass index; *MVPA* moderate to vigorous physical activity; *OR* odds ratio; *CI* confidence interval

## Discussion

In the current study, different trajectories of MVPA over one year were identified using LCGM, in a sample of young adults with mobility disability who participated in a physical activity intervention. The results indicated the presence of four distinct trajectories of MVPA. Three of the trajectories followed an increasing pattern, while the fourth one had a decreasing pattern and represented 28% of total participants. In addition, potential predictors of the trajectories were assessed, and it was found that those with younger age and higher baseline MVPA were more likely to have an increasing pattern of MVPA. Furthermore, it is worth mentioning that when looking at the total sample, in contrast to the subgroup patterns, MVPA seemed to remain stable over the one-year period.

Although the present analysis is based on interventional data, the number and characteristics of the identified trajectories cannot be attributed to the two interventions. This is mainly because all individuals were exposed to a physical activity intervention and there was no unexposed comparison group. If such a group were present, it would be possible to compare the number and shape of trajectories between exposed and unexposed individuals. Accordingly, potential differences in the trajectories could be interpreted as causal effects of receiving a physical activity intervention, under the condition that participants were randomly assigned to intervention and control groups. Moreover, if the sample size were large enough, it would also be possible to assess whether different trajectories occur between the mobile app and supervised program.

Regarding the interpretation of the current findings, any differences between the two groups during the follow up could be considered as intervention effects, due to the randomization. Therefore, the differences in frequencies of the trajectories between the mobile app and supervised groups could be attributed to the two interventions. More specifically, the mobile app group was more likely to follow the ‘Normal/Decrease’ and ‘High/Increase’ trajectories, whereas ‘Normal/Increase’ and ‘Normal/Rapid Increase’ trajectories were more frequent in the supervised group. Furthermore, the results could be generalized among individuals that have received a physical activity intervention, indicating that not all individuals that engage in physical training improve their MVPA levels and characteristics such as age and baseline MVPA levels might predict future patterns.

Very few studies have previously used a person-centered approach to evaluate intervention effects and even fewer have assessed changes in physical activity levels. Two previous studies have identified distinct trajectories of physical activity over one year period, using LCGM, in adults who received lifestyle recommendations (Imes et al., 2018; Pedersen et al., 2019). In one study, physical activity was objectively assessed as step counts per day and based on this outcome four distinct trajectory groups were identified (Imes et al., 2018). The two groups with fewer baseline steps followed a decreasing pattern, the one with the most steps had an increasing pattern, and the other one did not change (Imes et al., 2018). In the second study, three trajectories of self-reported physical activity scores were identified (Pedersen et al., 2019). The group with the lowest baseline levels improved, the one with moderate levels remained stable, and the one with the highest levels decreased (Pedersen et al., 2019). Only the first study assessed potential predictors of class assignment and found that BMI and self-reported general health were significant predictors (Imes et al., 2018). The high inconsistency in these findings, also considering the results of our study, can be explained by several factors. Primarily, the target population of all three interventions was different, one study targeted obese individuals, the other recruited manual workers, while the current study focused on individuals with mobility disability. Moreover, all three studies had different intervention components and the outcome was measured in different ways.

The biggest challenge when performing LCGM in intervention studies is limited statistical power to identify the true number of trajectories in a population, often due to small sample sizes and high loss to follow-up. It would be ideal to perform a sample size calculation before using latent class models, but such procedure is complicated and depends on multiple factors which are difficult to know a priori*,* such as the number of true trajectories or the distance between classes. Simulation studies suggest that larger sample sizes are required when the number of classes and missing data are increasing, and the distance between the classes and follow ups are decreasing, (Kim, 2012; Tein et al., 2013). Accordingly, in the best case a minimum sample size of 200 is required when the true number of classes is 2 and a sample size of about 1000 when the true number of classes is close to 5 (Kim, 2012; Tein et al., 2013). In the current study, low statistical power was partly addressed by performing multiple imputations for the missing values and by choosing LCGM which assumes that all people within a trajectory have the same slope and intercept, resulting in elimination of within-class variability (van der Nest et al., 2020). Another factor that was considered is the combination of fit criteria for determining the correct number of classes. Although there is not an agreement on which criteria should be preferred, simulation studies have shown that, in the presence of small sample sizes, BIC is more accurate compared to other information criteria (Nylund et al., 2007; Yang, 2006). Nevertheless, the results of the current study need to be interpreted with caution.

### Suggestions for future research

Regardless the challenges of using latent class trajectory analysis in intervention studies, this method has several implications for public health and future research. Most importantly it allows the identification of hidden patterns of change in a population and their determinants. This knowledge is valuable for the design of health promotion programs. For instance, it would be possible to include only people that could be benefited by a public health intervention. Moreover, future studies could focus on individuals that do not show improvements in the outcomes of interest after receiving interventions that have been effective in most of the population. Instead, different intervention components can be evaluated to better understand what could benefit these individuals. However, it needs to be highlighted that since interventions using latent class models are scarce in most fields, the findings need to be replicated in order to have these implications. In addition, it is strongly recommended that future intervention studies with a person-centered approach include larger sample sizes and more follow-up points in order to determine with higher certainty the correct number of latent classes and potential predictors of them. Finally, inclusion of a control group in such studies would enable the assessment of intervention effects on the number and characteristics of the trajectories.

### Conclusions

LCGM could be a useful method for analyzing intervention studies, as it allows the detection of different patterns of change in the outcomes of interest. Such approaches can help identifying individuals that are benefited by an intervention and why, which could promote the design of more targeted interventions in the future. As it was discussed in this paper, the main concern when using LCGM in intervention studies is the often insufficient statistical power to detect the correct number of trajectories. It is therefore recommended that future intervention studies using this method include larger sample sizes and provide a careful interpretation of their findings. Regardless the challenges that might accompany LCGM, researchers are encouraged to use this method for studying intervention outcomes.

## Electronic supplementary material

Below is the link to the electronic supplementary material.

Supplementary material 1 (DOCX 38 kb)

## Data Availability

The dataset analyzed during the current study is not publicly available but is available from the corresponding author on reasonable request.

## Code availability

The code generated during the current study is not publicly available but is available from the corresponding author on reasonable request.
