# Supplementary material for: Latent class growth modelling for the evaluation of intervention outcomes: example from a physical activity intervention
Source: J Behav Med. 2021 Mar 25;44(5):622–9. doi: 10.1007/s10865-021-00216-y (PMC8484241; doi:10.1007/s10865-021-00216-y)
Supplement: Supplementary file 1 — Supplementary material 1 (DOCX 38 kb) [file 10865_2021_216_MOESM1_ESM.docx]

ELECTRONIC SUPPLEMENTARY MATERIAL

for manuscript “Latent class growth modelling for the evaluation of intervention outcomes: example from a physical activity intervention”

Electronic supplementary figure 1

Electronic supplementary figure 2

Electronic supplementary table 1

Electronic supplementary table 2

Electronic supplementary figure 1. Participant flow chart

Electronic supplementary figure 2. Mean moderate to vigorous physical activity (min/day) at baseline, 6 weeks, 12 weeks, and 1 year in the observed and imputed values

| **Baseline characteristics** | **Attended** n=59 | **Dropouts**^1^ n=51 | **P-value** |
| --- | --- | --- | --- |
| Females, n (%) | 49(83) | 41(80) | 0.718 |
| Mobile app group, n (%) | 21(36) | 34(67) | 0.001 |
| Age (years), mean ± SD | 34.6 ± 5.9 | 35.6 ± 6.8 | 0.411 |
| BMI (kg/m^2^), mean ± SD | 26.3 ± 4.8 | 27.2 ± 6.2 | 0.410 |
| VO_2_ max (ml/kg/min), mean ± SD | 35.5 ± 8.2 | 35.7 ± 8.5 | 0.906 |
| MVPA (min/day), mean ± SD | 40.8 ± 18.6 | 48.5 ± 25.4 | 0.071 |
| Bodily pain (score), mean ± SD | 53.3 ± 19.4 | 47.7 ± 19.6 | 0.137 |
| Physical functioning (score), mean ± SD | 74.4 ± 12.6 | 71.2 ± 20.1 | 0.308 |

Electronic supplementary table 1. Baseline characteristics of those that attended and those that dropped out at the 1-year follow up, p-value from t-tests and chi-squared tests

^1^ There were 19 dropouts at 6 weeks and 22 dropouts at 12 weeks; Abbreviations: BMI= body mass index; MVPA= moderate to vigorous physical activity; SD= standard deviation

| **Number of latent classes** | **Polynomial form** | **BIC** | **% Participants**  **per class** | **Posterior probabilities** |
| --- | --- | --- | --- | --- |
| 1 | Linear | -1969.52 | 100 | NA |
|  | Quadratic | -1971.77 | 100 | NA |
|  | Cubic | -1973.11 | 100 | NA |
| 2 | Linear | -1943.16 | 61.5/38.5 | 0.92/0.90 |
|  | Quadratic | -1946.05 | 63.7/36.3 | 0.93/0.90 |
|  | Cubic | -1948.81 | 63.9/36.1 | 0.93/0.90 |
| 3 | Linear | -1939.36 | 40.1/35.9/24.0 | 0.88/0.84/0.88 |
|  | Quadratic | -1943.72 | 41.1/32.7/26.2 | 0.88/0.82/0.91 |
|  | Cubic | -1947.71 | 40.3/33.4/26.3 | 0.88/0.83/0.91 |
| 4 | Linear | -1940.74 | 27.8/28.4/27.9/15.9 | 0.86/0.82/0.85/0.88 |
|  | Quadratic | -1941.70 | 30.2/30.2/22.5/17.1 | 0.87/0.82/0.87/0.91 |
|  | Cubic | -1947.65 | 30.9/28.6/23.2/17.3 | 0.88/0.83/0.89/0.92 |
| 5 | Linear | -1946.01 | 12.4/35.0/20.1/22.2/10.3 | 0.82/0.82/0.80/0.85/0.86 |
|  | Quadratic | -1945.17 | 22.5/34.6/17.5/12.3/13.1 | 0.88/0.83/0.85/0.87/0.91 |
|  | Cubic | -1950.43 | 20.9/24.1/24.2/18.5/12.3 | 0.89/0.88/0.89/0.89/0.92 |

Electronic supplementary table 2. Latent class growth analysis parameters

Abbreviations: BIC= Bayesian information criteria
